# Reelin Together with ApoER2 Regulates Interneuron Migration in the Olfactory Bulb

**DOI:** 10.1371/journal.pone.0050646

**Published:** 2012-11-29

**Authors:** Sabine Hellwig, Iris Hack, Birgit Zucker, Bianka Brunne, Dirk Junghans

**Affiliations:** 1 Department of Psychiatry and Psychotherapy, University of Freiburg Medical School, Freiburg, Germany; 2 Institute of Anatomy and Cell Biology I, University of Freiburg, Freiburg, Germany; 3 Institute of Neuroscience and Medicine (INM-2), Research Center Jülich, Jülich, Germany; 4 Department of Neurology, Neurocenter, University of Freiburg Medical School, Freiburg, Germany; 5 Institute of Structural Neurobiology, Center for Molecular Neurobiology, Hamburg, Germany; Baylor College of Medicine, United States of America

## Abstract

One pathway regulating the migration of neurons during development of the mammalian cortex involves the extracellular matrix protein Reelin. Reelin and components of its signaling cascade, the lipoprotein receptors ApoER2 and Vldlr and the intracellular adapter protein Dab1 are pivotal for a correct layer formation during corticogenesis. The olfactory bulb (OB) as a phylogenetically old cortical region is known to be a prominent site of Reelin expression. Although some aspects of Reelin function in the OB have been described, the influence of Reelin on OB layer formation has so far been poorly analyzed. Here we studied animals deficient for either Reelin, Vldlr, ApoER2 or Dab1 as well as double-null mutants. We performed organotypic migration assays, immunohistochemical marker analysis and BrdU incorporation studies to elucidate roles for the different components of the Reelin signaling cascade in OB neuroblast migration and layer formation. We identified ApoER2 as being the main receptor responsible for Reelin mediated detachment of neuroblasts and correct migration of early generated interneurons within the OB, a prerequisite for correct OB lamination.

## Introduction

The OB is a phylogenetically old cortical region, which like the neocortex is laminated and consists of five individual layers: glomerular layer (GL), external plexiform layer (EPL), mitral cell layer (MCL), internal plexiform layer (IPL) and granule cell layer (GCL). These layers are formed in an inside-outside manner during development [1]. Distinct neuronal populations are found in the layers of the OB: mitral cells as principal projection neurons and in addition a morphologically similar but biochemically heterogeneous group of interneurons [2],[3],[4],[5]. Subsets of interneurons, such as granule cells, periglomerular cells and interneurons of the external plexiform layer, occupy distinct layers and are therefore distinguishable based on their position within the OB [3], [6].

The OB is one of the few structures in the mammalian central nervous system in which newly generated neurons are continuously produced throughout adulthood [7], [8]. At embryonic stage, interneurons have been shown to arise from the lateral ganglionic eminence and dorsal telencephalon [9], [10], [11], [12], [13], [14] whereas postnatally interneurons derive from the anterior part of the subventricular zone (SVZ) of the lateral ventricle [15], [16], [17], [18]. From this neurogenic region, precommitted neuroblasts migrate tangentially in a chain-like organization into the OB, forming the rostral migratory stream (RMS). After entering the OB, neuroblasts detach from the RMS, switch from chain to radial migration and ascend radially into the defined layers where they finally mature. The interaction of radial migrating cells with their environment and the molecular signals underlying and regulating this process are as yet poorly understood (reviewed in [19]).

One key signaling pathway known to orchestrate migration and layer formation involves the extracellular matrix protein Reelin (reviewed by [1], [20], [21], [22], [23], [24]). Canonical Reelin signaling is mediated by the two membrane bound lipoproteinreceptors apolipoprotein E receptor 2 (ApoER2) and very low density receptor (Vldlr) [25], [26], [27] and the cytoplasmatic adapter protein disabled 1 (Dab1) [27],[28],[29],[30],[31]. Binding of Reelin to one of its receptors induces phosphorylation of Dab1 essential to trigger downstream signaling [26], [27], [32]. Null mutations in the *Reelin* or *Dab1* genes as well as *Vldlr:ApoER2* double-null animals result in *reeler*-like phenotypes characterized by severely altered cortical layering [27], [28], [30], [31], [33]. Several modes of Reelin action have been identified so far. Reelin can act as stop signal [34], chemoattractant [35] and in addition as detachment signal for migrating neurons [36], [37]. However, which of these functions are responsible for correct cortical layering is heavily debated.

Reelin is prominently expressed in the OB [38] and abnormalities in the OB of *reeler* mice have been described previously including a size reduction as well as morphological alterations in the periglomerular, mitral and granule cell layers [36], [39]. Reelin also acts as a detachment signal in the OB by inducing the switch from tangential oriented chain-migration of RMS neuroblasts to radial migration [36]. However, studies on ApoER2, Vldlr, Dab1 and Reelin deficient animals concluded that chain migration of neuroblasts within the RMS and SVZ might be independent of Reelin [40]. Nevertheless, the precise role of Reelin signaling components in OB layering and migration remains elusive.

In the present study we aimed to address this question. Therefore, we examined the integrity of the RMS and the detachment and lamination process in the OB of *Vldlr* ^−/−^, *ApoER2* ^−/−^ single and double knockouts, *Dab1^−/−^* and *reeler* mutants by analyzing precursor migration in a matrigel explant culture system, BrdU pulse labeling experiments and in addition immunhistochemical analysis of OB layer formation and cell positioning at different developmental time-points. In this context, we demonstrate that ApoER2 is the main receptor mediating the detachment signal of neuroblasts and correct migration of early generated interneurons. The comparison of *reeler* and *Dab1*-mutants also suggested that non-canonical Dab1-independent Reelin signaling might play a role in OB layer formation.

**Figure 1:**
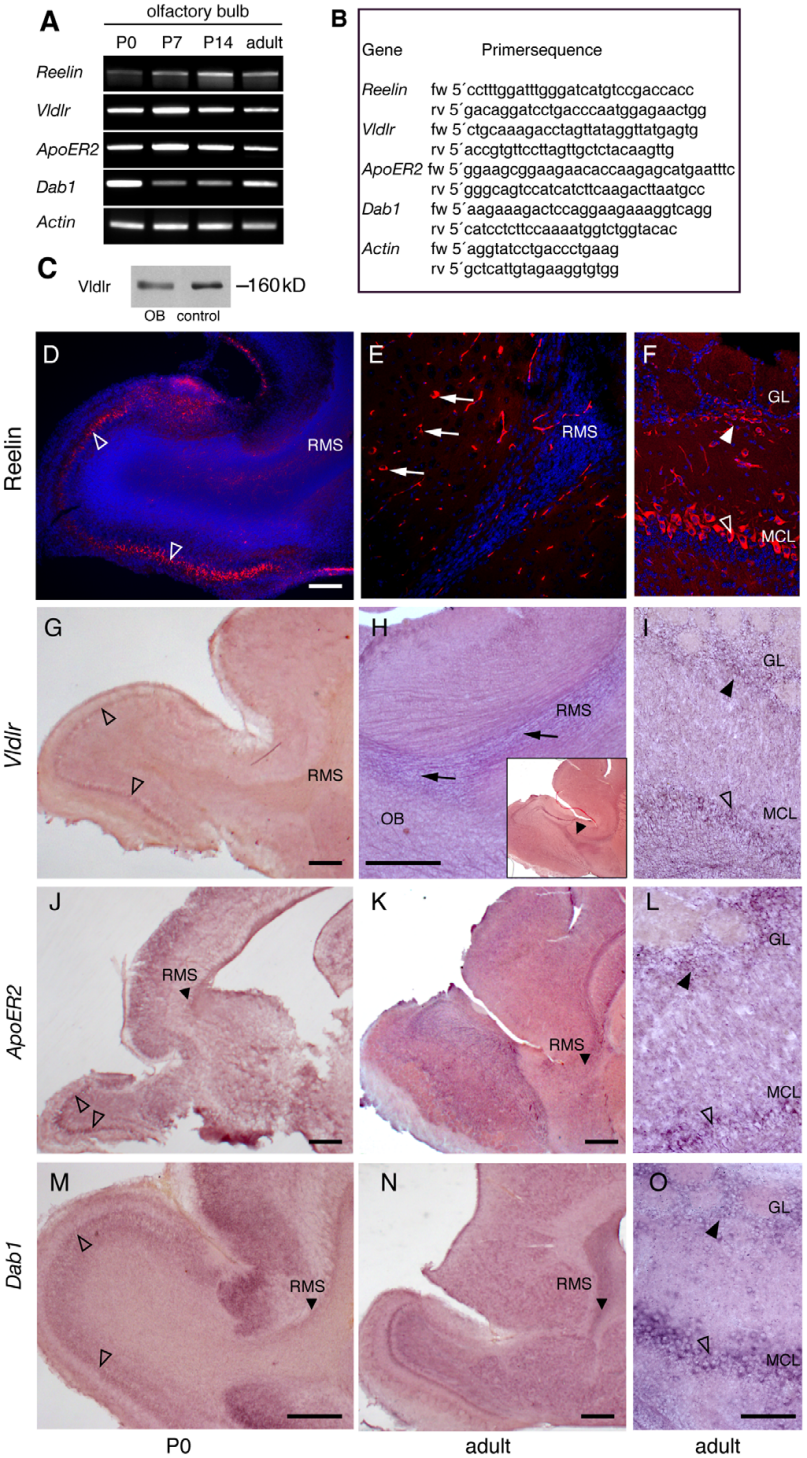
Expression of Reelin and components of its signaling pathway in the forebrain. (A) RT-PCR analysis at P0, P7, P14 and adult age revealed *Reelin*, *ApoER2*, *Vldlr* and *Dab1* mRNA expression in the OB. Samples were normalized to *β-actin* expression. (B) RT-PCR primers sequences (C) Western immunoblot analysis of adult OB tissue and transfected control fibroblasts confirming protein in the OB. (D-F) At P0 strong Reelin expression is found in the mitral cell layer of the OB (open arrowheads, D). Adult mice express Reelin in the MCL (open arrowhead) and in a periglomerular position (arrowhead, F). Reelin positive cells (arrows) are also seen in close vicinity to the rostral migratory stream (RMS, E). Migrating precursors in the RMS are negative at both analyzed timepoints. (G-I) Early postnatal *Vldlr* expression is restricted to the MCL (open arrowhead, G). At adult age *Vldlr* mRNA is found in the RMS (arrows, H and insert, arrowhead), in the MCL (open arrowheads, I) and in the innermost part of the glomerular cell layer (GL), (arrowheads, I). Similar expression patterns were obtained by in situ hybridization for *ApoER2* (K-L) and *Dab1* (N, O) on sagittal sections of adult wt forebrains. Of note, both are expressed in the RMS at P0 (J, M) and migrating precursors. Scale bars: D, G, H, J, K, M, N: 200 µm; E, F, I, L, O: 100 µm.

## Materials and Methods

### Ethics Statement

Experiments were performed in agreement with the German law on the use of laboratory animals and institutional guidelines of the University of Freiburg. The use of animals was approved by “Regierungspräsidium Freiburg (Freiburg regional council)” and the animal welfare office of the University of Freiburg Ref. No. X-08/04A. All animal experiments were performed at the University of Freiburg, Dept. of Neuroanatomy.

**Figure 2:**
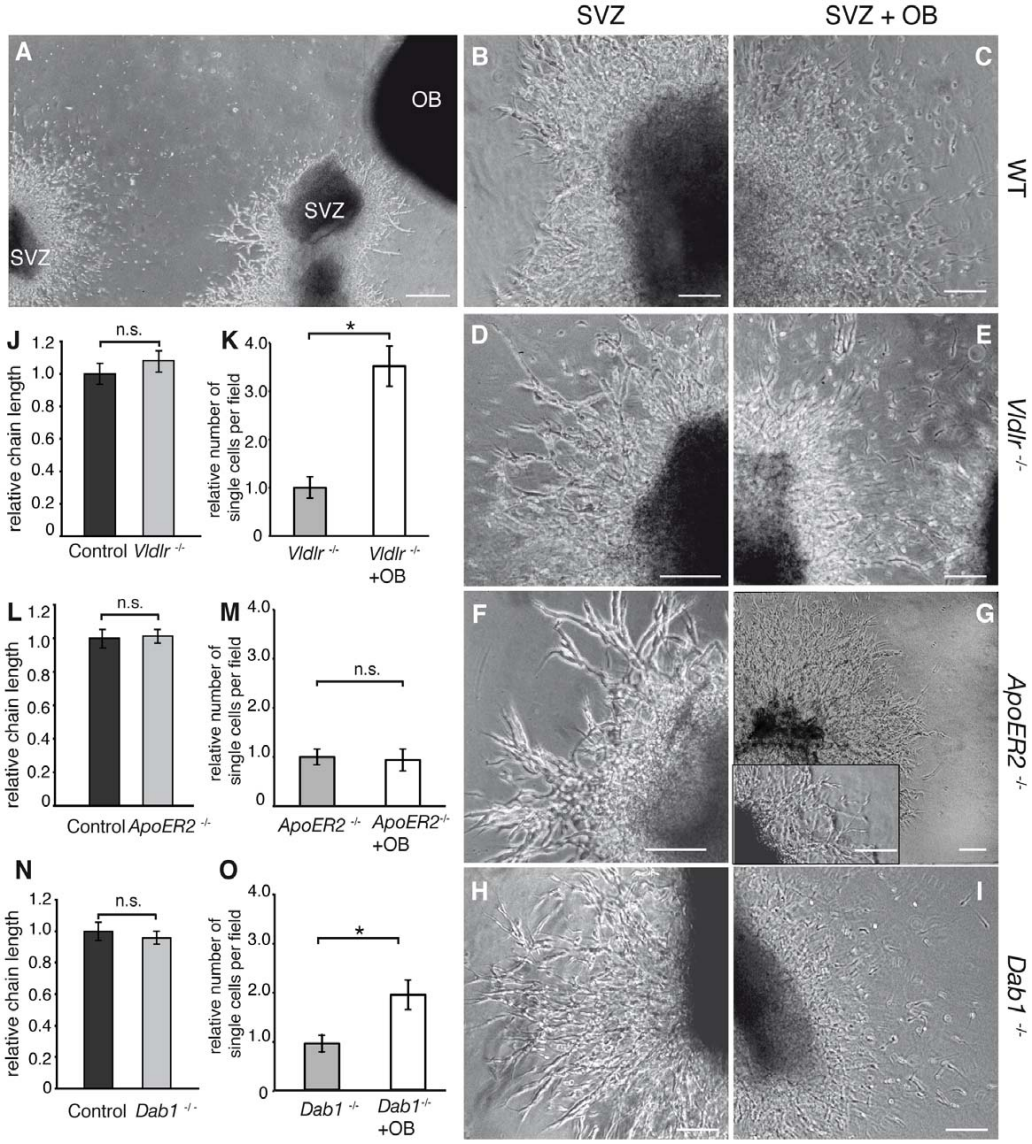
The Reelin detachment signal in vitro is mediated by ApoER2. (A) Experimental setup: co-culture of OB and explants from the subventricular zone (SVZ). After 48 hours all analyzed explants showed radial chain migration when cultured without OB. No differences were observed comparing the analyzed mutants with control animals (wild-type (wt, B), *Vldlr^−/−^* (D), *ApoER2^−/−^* (F) and *Dab1^−/−^* (H) ). Co-culture of SVZ explants and OB induced detachment of neuroblasts from the explant in wt mice (C) and *Vldlr^−/−^* (E) and *Dab1^−/−^* mutants (I). SVZ-cultures from *ApoER2^−/−^* mice (G) remained unaffected (insert: higher magnification of another *ApoER2^−/−^* explant). (J, L, N) Quantification of the relative migration distance (K, M, O) Quantification of the relative number of single cells per field. Statistical analysis by using Wilcoxon-Mann-Whitney test with *indicating significant difference. Data expressed as mean ± SEM. Scale bars: A: 200 µm; all others: 50 µm.

### Animals

*Reeler* mice were maintained on a B6/C3Fe background. *ApoER2^−/−^*, *Vldlr^−/−^* single and double mutant mice as well as *Dab1^−/−^* mutants were maintained on a mixed 129SvEv × C57BL/6J background. The day of birth was considered postnatal day (P) 0.

### RNA Preparation and RT-PCR

For cDNA synthesis total RNA was isolated from OBs of P0, P7, P14 and adult wild-type mice and *ApoER2^−/−^* mutant animals (n = 2–4 per genotype and developmental time point) using a tissue homogenizer and Trizol reagent (Invitrogen). Total RNA was DNase 1 treated (30 min, Roche Diagnostics) and purified (RNeasy mini kit, Qiagen). cDNA synthesis was performed using random primers (Promega) and Superscript II reverse transcriptase (Invitrogen). Dilution series were made and PCR performed with primers designed against β-actin for each individual cDNA to equalize template cDNA concentrations. All PCRs were performed by using junction primers specific for the individual members of the Reelin signaling cascade (Fig. 1B). All samples were tested on genomic and cDNA to confirm specificity and purity of the template. Individual PCR reactions were run in parallel and repeated at least three times.

**Figure 3:**
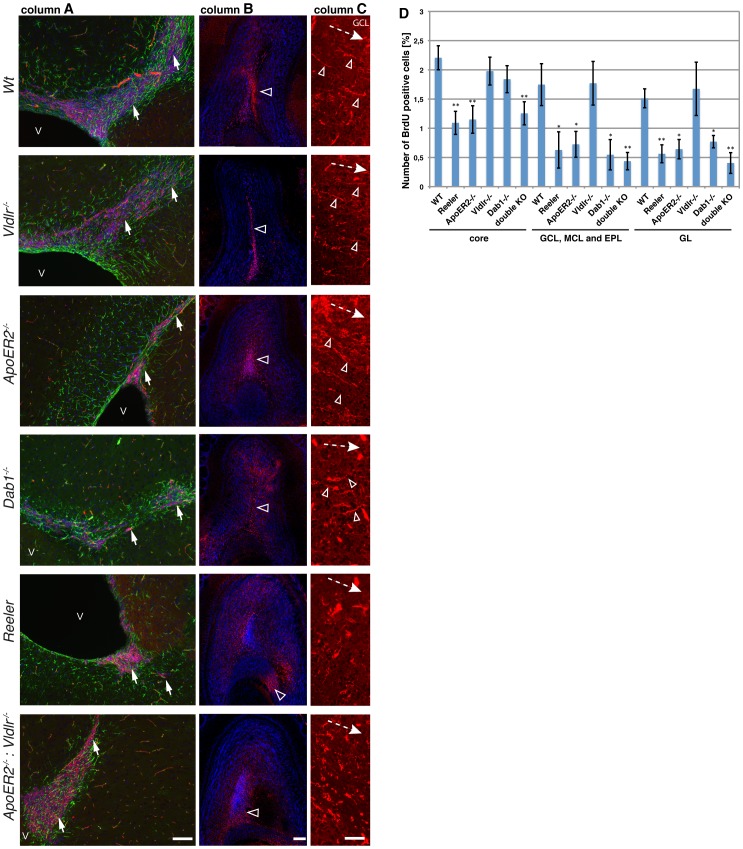
Functional integrity of the rostral migratory stream (RMS) and detachment process in vivo. Immunohistochemistry for PSA-NCAM (red), GFAP (green) and DAPI on sagittal sections through the forebrain of adult wild-type mice, *Vldlr* ^−/−^, *ApoER2* ^−/−^, *Dab1* ^−/−^, *reeler* and ApoER2^−/−^:*Vldlr^−/−^* mutant animals show the presence of neuroblasts proximal to the SVZ (arrows) (column A) and approaching the OB (open arrowheads) (column B) in all animals analyzed. Higher magnification on frontal sections and PSA-NCAM staining revealed a proper detachment of neuroblasts with the typical switch from chain migration to radial ascension into layers in wild-type mice, *Vldlr* ^−/−^ and *ApoER2^−/−^* mutants (column C). Single cells extend their leading process towards outer layers (open arrowheads, dashed arrow indicates migration direction from the RMS towards distal/upper layers). In contrast, *reeler* mutants as well as double receptor knockout mice exhibit a severe impairment of the detachment process (column C). Dab1^−/−^ animals appear to have only a minor phenotype (arrowheads). In (**D**) the number of BrdU labeled nuclei 16 days after BrdU injection in different areas of the OB (core (proximal to the RMS), GCL (granule cell layer), MCL (mitral cell layer) and EPL (external plexiform layer) area and GL (glomerular cell layer) are shown. Numbers are given as BrdU^+^/total cells [%]. Student′s test compared number of BrdU^+^ in mutants to corresponding region of wild-type animals. *p<0.05; **p<0.01. V = ventricle; Scale bars: column A 100 µm, column B 200 µm and column C 50 µm.

### Real Time-PCR

Real time-PCR (Q-PCR) studies were performed with a Bio-Rad iCycler by using SYBR-Green PCR Master Mix (Applied Biosystems, Darmstadt, Germany) through 50 PCR cycles (95°C for 30 s, 57°C for 60 s, 72°C for 90 s). Each cDNA sample was run in triplicates for the target and the normalizing gene (ß-actin) in the same 96-well plate. Specificity of amplicons was determined by melt curve analysis and gel electrophoresis. Sequences of used junction primers are listed in Fig. 1B.

**Figure 4:**
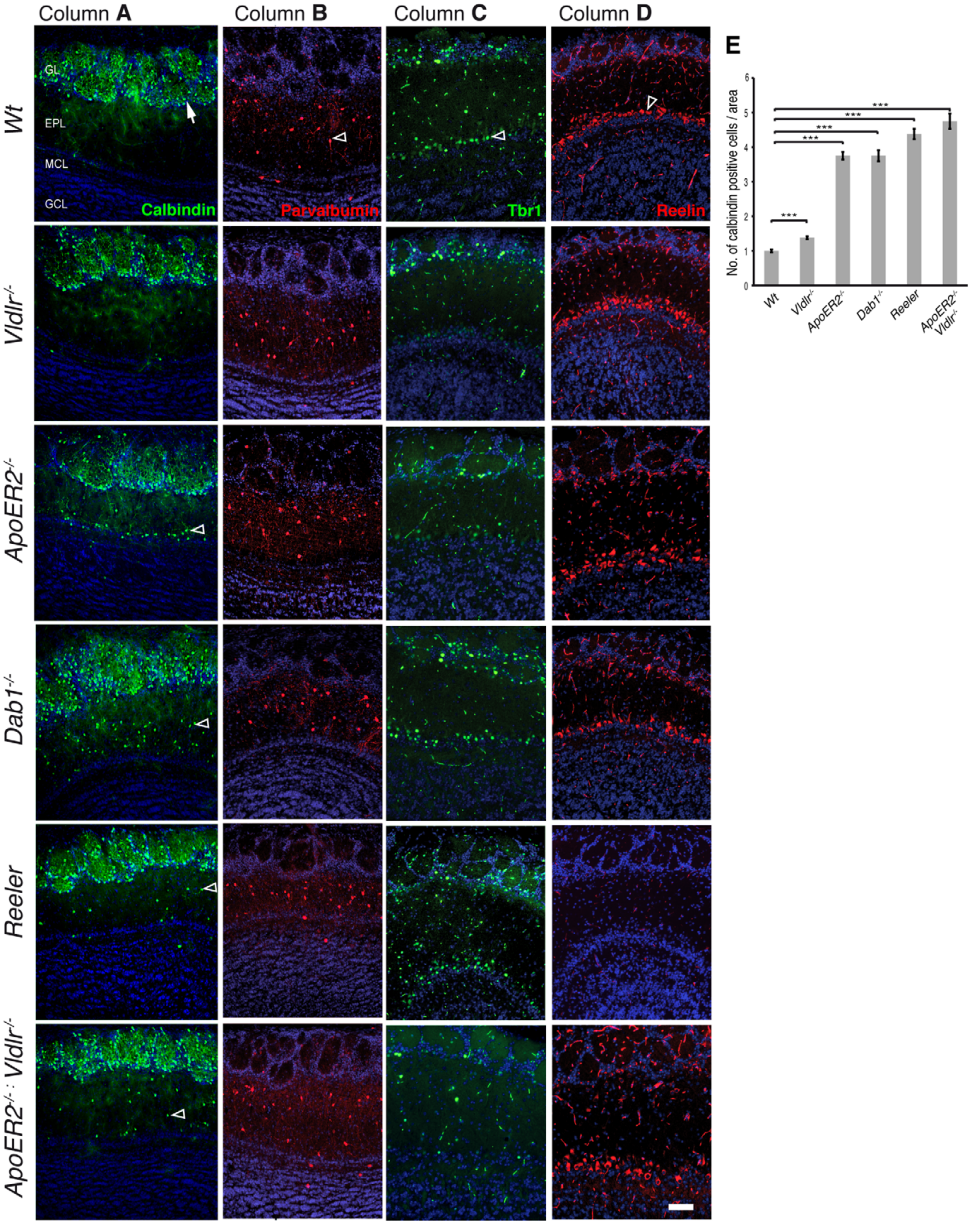
Labeling of early generated interneurons. Immunohistochemistry for Calbindin on frontal sections through the OB of adult wild-type mice showed strong expression in the glomerular cell layer (GL, column A, wt). A similar staining pattern was observed in *Vldlr^−/−^* mice (column A, *Vldlr^−/−^*) although few positive cells hosted in the external plexiform layer. *ApoER2^−/−^*, *Dab1^−/−^*, *reeler* and *ApoER2* ^−/−^:*Vldlr^−/−^* mutants show clearly two separate cell populations using Calbindin as marker (column A). A superficial one in the typical periglomerular position and a deep one in the EPL (arrowheads, column A). (Column B) Immunohistochemistry for Parvalbumin on frontal sections through the OB of adult mice labeled interneurons in the external plexiform layer (EPL). No differences were observed for the analyzed mutants compared to wild-type mice. Staining for Tbr1 (column C) and Reelin (column D) as markers of the MCL showed no alterations in the analyzed animals except the loss of Reelin immunoreactivity in *reeler* mutants. Quantification of the relative number of Calbindin positive (E) cells per area in the EPL after normalization to the wt situation (n = 3−5 animals per genotype). Wilcoxon-Mann-Whitney test; Data expressed as mean ± SEM. Scale bar 100 µm. MCL, mitral cell layer; GCL, granule cell layer.

#### Data analysis and statistics

Expression of the mRNA of interest in each sample was calculated for Q-PCR by normalization of Ct values to the reference RNA (ß-actin) using the equation: V = (1+E reference) (Ct reference)/(1+E target) (Ct target) in order to correct for potential differences in PCR amplification efficiencies. V = relative value of target gene normalized to reference (ß-actin), E = PCR amplification efficiency, Ct = threshold crossing cycle number [41]. Differences between genotypes were assessed using an unpaired, two-tailed Student’s t-test.

### Immunoblotting

OB tissue of wild-type animals and *Dab1* mutant mice (3 month of age) was homogenized in ice-cold lysis buffer (20 mM Tris-HCl, 0.15 M NaCl, 2 mM EDTA, pH 7.5, protease inhibitor mixture; Roche Diagnostics). Lysates were cleared two times by centrifugation (10,000 g, 5 min, 4°C). The soluble cytosolic fraction was removed after ultracentrifugation (200,000 g, 30 min, 4°C) and pellets resolved in lysis buffer containing additional 0.1% sodium dodecyl sulfate and 1% Trition-X-100. Soluble membrane fractions were obtained by a second ultracentrifugation step (100,000 g, 30 min, 4°C) and analyzed by standard SDS-PAGE and Western immunoblotting using chemoluminescence detection techniques (Super Signal West Pico, Perbio Science). Specificity of Vldlr antibodies was confirmed by loading cell lysates of NIH 3T3 fibroblasts transfected to express Vldlr-Dab1-GFP-fusion protein [42].

**Figure 5:**
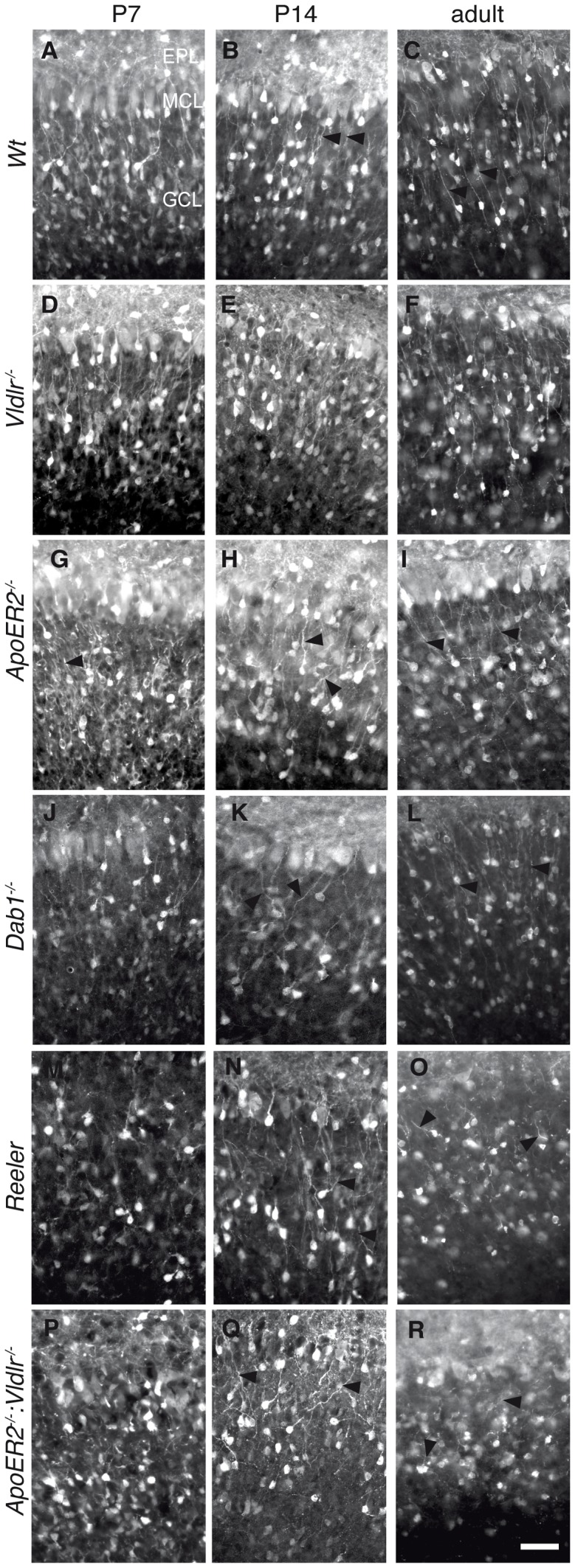
Labeling of Calretinin-positive interneurons in the granule cell layer (GCL) at different developmental timepoints. Immunostaining for Calretinin on frontal sections through the OB. Wild-type (A-C) and *Vldlr ^−/−^* mice (D-F) at P7 and P14 and adult age reveal the typical formation of Calretinin positive interneurons in the GCL, with a radial orientation of neurites towards upper layers (arrowheads). In *reeler* (M-O) and *ApoER2^−/−^:Vldlr^−/−^* double KO animals (P-R) a severe disruption of the GCL is observed. Calretinin-positive interneurons accumulate in clusters and the radial orientation of dendrites is lost (arrowheads). In *ApoER2^−/−^* mutants a *reeler*-like phenotype is found one week after birth (G). However, at P14 a compact layer begins to form with few cells extending their neurites towards the surface (H), while adult ApoER2^−/−^ mice (I) exhibit minor alterations compared to wild-type mice. *Dab1^−/−^* animals (J-L) exhibit a weak *reeler* like phenotype at P7 and P14 with less strict orientation of neuritis towards upper layers (arrowheads, J and K). At adult stage *Dab1^−/−^* mice appear similar as wild-type animals (L). Scale bar: 50 µm. and *Dab1^−/−^* mice (J-L).

For immunoblotting the following primary antibodies were used: anti-Vldlr (1∶1,000, a kind gift from A. Goffinet), anti-Crmp-1 (1∶1,000, AB9216, Millipore) and anti ß-actin (1∶5,000, A2066, Sigma-Aldrich). Secondary antibodies: horseradish peroxidase (HRP) linked anti-mouse (1∶10,000, NA931V, Amersham Biosciences) and HRP linked donkey anti-rabbit (1∶10,000, NA934V, Amersham Biosciences).

### In situ Hybridization

#### Template DNA

Total RNA from P0 wild-type brain was reverse-transcribed using reverse transcriptase (Superscript II, Invitrogen) and used as a template for PCR reactions.

The following oligonucleotide primers containing additional T3/T7 binding sites were used to amplify a specific 330 BP Vldlr DNA fragment: fwd 5′-GCAGATGAGTTCACTTGCTCC-3′ and rev 5′-CCTTGCAGTCAGGGTCTCC-3′). The PCR product was purified and sequenced before in vitro transcription. For other templates plasmid DNA was linearized with the appropriate enzyme and purified.

#### In vitro transcription

*In vitro* transcription was performed using 1 µg linearized plasmid DNA or PCR template in the presence of digoxigenin-11-UTP (DIG labeling mix, Roche), RNasin (Promega), 1× transcription buffer and T3, SP6 or T7 RNA polymerase (Roche Diagnostics). The following DIG-labeled riboprobes were generated: Dab1 [43], ApoER2 [44], Crmp-1 (see Allen Brain Atlas, http://mouse.brain-map.org, Ref. 73512561) and Vldlr.

#### RNA detection by in situ hybridization

Adult wild-type brains were fixed in freshly prepared 4% PFA in PBS, cryoprotected in 30% sucrose and frozen in isopentane at −60°C. *In situ* hybridization was performed on 50 µm free-floating cryostat sections as described previously [45].

### Matrigel Culture

Cultures of SVZ-explants (n = 3–5 for each genotype) were performed as described previously [36]. P2 brains were dissected and the OBs separated. The forebrains were sliced frontally into 400 µm sections in ice cold Hank′s balanced salt solution (HBSS, Invitrogen) using a vibratome (Leica). From appropriate sections the SVZ was dissected out and cut into smaller pieces (100–300 µm in diameter). After mixing the SVZ-explants with Matrigel (Becton Dickinson) and incubation at 37°C for 10 min the polymerized gel was covered with 500 µl nutrition medium (Neurobasal A, 2% B27 supplement, 50 U/ml penicillin, 50 µg/ml streptomycin, 0.5 mM L-glutamine, Invitrogen). For culturing SVZ-explants uncoated glass cover slips (12 mm) were placed into non-treated 4 well dishes (179820, Thermo Fisher Scientific). For co-culturing experiments pieces of OB (approximately 300 µm diameter) were placed next to the SVZ-explants before polymerization of the matrigel. The cultures were maintained in a humidified atmosphere with 5% CO_2_ at 37°C for 2 days. After 48 h explants were monitored and the number of individual neurons per field and the length of migratory chains were measured using AnalySIS image analysis software (SIS). Statistical analysis was performed by using the non-parametric Wilcoxon-Mann-Whitney Test. Significance was assigned for all tests at *p*<0.05.

### Immunohistochemistry

#### Staining procedure

Brains were fixed in 4% PFA in PBS and sliced sagittally on a vibratome (50 µm). Sections were pre-incubated for 60 minutes in blocking solution (10% fetal calf serum (FCS) in 1× PBS) at room temperature. Subsequently, sections were incubated with the primary antibodies in 3% FCS containing 0.1% Triton-X 100 in 1× PBS overnight at 4°C. For staining against PSA-NCAM Triton-X 100 was avoided. After washing (3×10 min, PBS) at room temperature, sections were incubated with secondary fluorochrome-conjugated antibodies (Alexa-Fluor series, Invitrogen) for 2 hours at room temperature. After rinsing in PBS (three times, 30 min) sections were mounted in fluorescent mounting medium (DAKO) supplemented with 1 µg/ml 4′, 6-Diamidin-2-phenylindol (DAPI).

#### Antibodies

The following antibodies were used: anti-Reelin (1∶250, Millipore, MAB5364); anti-Calbindin, anti-Calretinin and anti*-*Parvalbumin (each 1∶1,000, Swant, CB-38a, 7699/4, PV25), anti-BLBP (1∶500, Millipore, AB9558), anti-Polysialic acid (1∶200, Millipore, MAB5324), anti-GFAP (1∶1,000, DAKO, Z0334), rabbit anti-Tyrosin Hydroxylase (1∶1,000, Millipore, AB152), and anti-Tbr1 (1∶5,000, Millipore, AB9616). Secondary antibodies: Alexa-Fluor series (Invitrogen: A-11004, A-11008, A-11011, A-21043, each antibody 1∶1000).

#### Quantification and statistical analysis

The number of Calbindin-positive interneurons was obtained from 10 section per OB (n = 3−4 for each genotype), respectively. Cell number was estimated in the external plexiform layer. The area of interest was measured by using AnalySIS- software (SIS). Statistical analysis was performed by using the non-parametric Wilcoxon-Mann-Whitney Test. Significance was assigned for all tests at *p*<0.05.

### BrdU Injection and Staining

Six to eight weeks old mice (n = 3−5 animals of each genotype) were injected intraperitoneally with a sterile solution of BrdU (50 mg/kg, Roche Diagnostics) in PBS. Brains were fixed in freshly prepared 4% paraformaldehyde (PFA) in PBS in paraffin embedded and cut into 7 µm serial sagittal sections using a microtome (Leica). For BrdU detection sections were de-paraffinized, antigen-recovery protocols using acidic and alkaline treatment applied and sections stained as previously described [46]. A monoclonal mouse anti-BrdU antibody (1∶1,000, Roche Diagnostics) was used to detect BrdU incorporation. Sections were exposed to secondary antibodies (Alexa-Fluor series, Invitrogen) and finally embedded in fluorescent mounting medium (DAKO).

The number of BrdU-positive cells was counted on eight sagittal sections through the OB and RMS per genotype. The OB was divided into three areas (core, medium incl. GCL, MCL and EPL and finally the glomerular cell layer). Of each area on each section 200–400 cells (DAPI positive nuclei) were counted and the number of BrdU positive cells determined and expressed as number of BrdU positive cells per 100 total cells.

## Results

### Expression of Reelin, ApoER2, Vldlr and Dab1 in the OB

We analyzed mRNA expression of *Reelin* and components of its signaling pathway in the OB by semi-quantitative RT-PCR at different developmental time points (P0, P7, P14 and adult). *Reelin* mRNA was present in OB samples at all developmental stages analyzed (Fig. 1A). Similar results were found for *Vldlr*, *ApoER2* and *Dab1* (Fig. 1A). Western immunoblotting analysis confirmed the presence of Vldlr protein in the adult OB of wild-type mice (Fig. 1C).To study the corresponding expression patterns, we performed immunohistochemistry for Reelin and in situ hybridization experiments for *ApoER2*, *Vldlr* and *Dab1* on sagittal brain sections of early postnatal (P0) and adult wild-type mice. At P0 Reelin was strongly expressed in the MCL likely by mitral cells (Fig. 1D and F) whereas Reelin positive interneurons were detected in the innermost part of the GL of adult animals (Fig. 1F). At both time-points Reelin was not present in the RMS (Fig. 1D, E). However, some Reelin-positive cells were observed in close proximity to the RMS (Fig. 1E).

At early postnatal stages weak expression of *Vldlr* was observed in the MCL while the RMS remained negative (Fig. 1G). Of note, in the adult animal migrating neuroblasts in the RMS were *Vldlr* positive (Fig. 1H). In the adult OB *Vldlr* (Fig. 1I), *ApoER2* (Fig. 1L) and *Dab1* (Fig. 1O) expression were present within the MCL and in a periglomerular position. *ApoER2* and *Dab1* were expressed in the mitral cell layer at P0 and by migrating neuroblasts in the RMS at both developmental time points (Fig. 1J, K, M, and N).

### The Reelin Detachment Signal is Mediated via ApoER2

Reelin induces dispersal of chain migrating neuroblasts from SVZ-explants [36]. To investigate in more detail if components of the Reelin signaling cascade participate in the detachment process we cultured SVZ-explants of control animals, *Vldlr* ^−/−^, *ApoER2* ^−/−^ and *Dab1* ^−/−^ animals in a three dimensional extracellular matrix (Matrigel). This set up allows the analysis of chain migrating neuroblasts in vitro in the presence or absence of OB tissue, known to secrete Reelin. After 48 hours of culturing, all explants showed symmetrical radial chain migration of neuroblasts when cultured without OB tissue (Fig. 2B, D, F, H). In contrast, SVZ explants of wild-type (Fig. 2C), *Vldlr* ^−/−^ (Fig. 2E) and *Dab1^−/−^* mutants (Fig. 2I) cultured in the presence of OB tissue rarely showed migration of neuroblasts in chains. Precursor cells that migrated out of the explants dispersed and lost contact with the explant or with other cells. Importantly, co-culturing of SVZ tissue from *ApoER2^−/−^* mutants (Fig. 2G and insert) with OB tissue did not affect the radial chain migration. For quantification of the chain length of the migrating neuroblasts and the effect on dispersion induced by the presence of Reelin secreting OB tissue see (Fig. 2J–O).

These results indicate that the detachment signal in explant cultures requires ApoER2 and, unexpectedly, this process appears to be independent of Vldlr and Dab1.

### All Mutant Animals Exhibit an RMS and Show BrdU Incorporation into the OB

We examined the RMS in the various mutant animals as a prerequisite for correct interneuron turnover in the OB. We performed first immunohistochemistry for PSA-NCAM a marker of neuroblasts on sagittal sections of adult brains of wild type and mutant animals (Fig. 3). We found that all animals analyzed exhibited PSA-NCAM positive neuroblasts proximal to the SVZ (Fig. 3 column A) as well as distal when the RMS enters the OB (Fig. 3 column B). Next, to analyze whether neuroblasts actually enter the OB, we performed BrdU pulse labeling experiments to label newborn cells and analyzed adult mice 16 days after a single BrdU injections. We found BrdU immunoreactivity located within the OB in all analyzed mutant and wild-type animals revealing a functional RMS. BrdU positive cells were found in the core region at intermediate positions (GCL, MCL and EPL area) as well as in the GL of each analyzed OB (pictures not shown). Quantifications of BrdU positive cells and statistical analysis are shown in Fig. 3D. Of note, the *reeler* mutant as well as the *ApoER2^−/−^* and *ApoER2^−/−^:Vldlr^−/−^* double receptor knockout mutant mice showed a marked reduction in BrdU positive cells in all OB regions. In contrast, *Vldlr^−/−^* mutant mice exhibited BrdU incorporation within the OB similar to wild-type animals. Interestingly, *Dab1^−/−^* animals showed no reduction in BrdU staining in the core region of the OB and a significant reduction in outer layers. These data substantiate the findings of our culturing experiments that ApoER2 is the main receptor for Reelin mediated detachment of neuroblasts from chain migration into radial migration independent of Vldlr.

### Detachment of Neuroblasts in the Adult OB of ApoER2 Mutants is not Altered

Our in vitro data point towards ApoER2 as being the main receptor in the detachment process of neuroblasts within the OB. Therefore, we studied next the detachment process in vivo by performing immunohistochemistry for PSA-NCAM on frontal sections of adult OBs. We observed the characteristic switch from tangential to radial migration associated with the presence of a characteristic leading processes oriented towards OB layers in wild-type animals, *Vldlr^−/−^* and *ApoER2^−/−^* mutants (Fig. 3 column C). In contrast, *reeler* mutants show a severe impairment in the switch from chain to radial migration, which occurred to be phenocopied in *ApoER2* ^−/−^:*Vldlr^−/−^* double receptor knockout mutants (Fig. 3 column C). In these mutants leading processes were not oriented, hardly visible and the cells appeared not organized. Dab1 deficient mutants showed an intermediate phenotype. Here, the leading processes were often visible but not as strictly oriented towards outer layers and the cells appeared less organized than in wild type mice. However, the *Dab1* ^−/−^ phenotype was not as strong as in *reeler* and double-knockout receptor mutants.

### Formation of OB Layers in Mutants of the Reelin Signaling Cascade

To elucidate the role of Reelin and components of its signaling cascade in the formation of OB layers we used interneuron and mitral cell markers to examine their position within the OB of wild type, *Vldlr^−/−^*, *ApoER2^−/−^*, *Dab1^−/−^*, *reeler* and *ApoER2^−/−^:Vldlr^−/−^* double mutant animals.

#### Glomerular cell layer (Calbindin and Tyrosine Hydroxylase)

The calcium binding protein Calbindin is expressed by distinct interneuron populations of the glomerular cell layer. Thus, in wild-type mice Calbindin-positive somata are found in a periglomerular position. Cells extended their dendritic processes to form the characteristic spherical structure of a glomerulus (arrow, Fig. 4 column A). Only a few cells were distributed throughout other layers. A similar normal distribution of Calbindin-positive interneurons was observed in *Vldlr^−/−^* mutants (Fig. 4 column A) although a few but statistical significant number of Calbindin-positive interneurons were found in the EPL (see also Fig. 4E). In contrast, reeler, ApoER2*^−/−^*, *Dab1^−/−^* and *ApoER2* ^−/−^:*Vldlr^−/−^* mutant animals exhibited a more dramatic phenotype with the appearance of many mispositioned Calbindin positive cells in the EPL. In *ApoER2^−/−^* mutants the majority of the Calbindin-positive interneurons were located correctly in the GL. However, a large subset of mispositioned interneurons was found in the inner most part of the EPL adjacent to mitral cells (arrowheads, Fig. 4 column A, *ApoER2^−/−^*). Dab1-deficient animals, *reeler* mutants and double receptor knockout mice showed similar alterations in Calbindin-positive interneuron distribution compared to *ApoER2^−/−^* mice (arrowheads, Fig. 4 column A). Quantification of the mispositioned Calbindin-positive interneurons revealed that *ApoER2^−/−^* single and *ApoER2* ^−/−^:*Vldlr^−/−^* double knock out, *Dab1^−/−^* and *reeler* mutants exhibited the most pronounced phenotype regarding misplaced cells within the MCL compared to wild type and *Vldlr^−/−^* mutants (Fig. 4E). We also analyzed Tyrosine Hydroxylase (TH) positive cells representing a second distinct interneuron population located within the GL. Similar to Calbindin-positive interneurons the TH-positive population exhibits localization to the GL in wild type animals and mispositioning of cells in the EPL in *ApoER2^−/−^* single and *ApoER2* ^−/−^:*Vldlr^−/−^* double knock-out animals (Fig. S3A). The quantification of mispositioned TH positive interneurons revealed no aberrant changes in the EPL of *Vldlr^−/−^* mutants compared to wt animals.

#### External plexiform layer (Parvalbumin)

The analysis of Parvalbumin-positive cells of the EPL (mostly van Gehuchten cells) revealed no alterations in all mutant animals analyzed. All mutants exhibited the appearance of Parvalbumin positive interneurons in the EPL as seen in wt animals (arrowheads Fig. 4 column B).

#### Mitral cell layer (Reelin and Tbr-1)

Mitral cells as principal projection neurons are the major source of Reelin expression in the OB. Accordingly, Reelin served as marker for this cell population. Despite the lack of Reelin expression in the mitral cell layer in *reeler* mutants no alterations were found when analyzing adult receptor single and double knockout mice as well as Dab1 deficient mutant animals when compared to wild-type animals (Fig. 4 column D). Tbr-1 was used as additional mitral cell marker revealing a comparable distribution in the MCL in all mutant animals as seen in wild type (Fig. 4 column C).

#### Granule cell layer: Calretinin

The calcium binding protein Calretinin is expressed by subsets of interneurons in the GCL and GL of the OB [47]. Here it served as marker to examine the morphology of the GCL at different developmental time points (P7, P14, adult). In wild-type animals the Calretinin-positive interneurons of the granule cell layer show a compact organization with strictly oriented neurites projecting towards the superficial layers at all time-points analyzed (Fig. 5A–C with arrowheads labeling neurites). In contrast, Calretinin immunostaining of *reeler* mutants revealed a severe disruption of the GCL (Fig. 5M–O). Many Calretinin-positive interneurons appeared less confined to a particular region of the GCL and were distributed over the entire GCL, formed clusters and were often lacking extending dendrites and radial orientation of dendrites towards the EPL. The severity of this phenotype increased with age. A similar a *reeler*-like phenotype was observed in *ApoER2* ^−/−^:*Vldlr^−/−^* double-knockout mice with clusters and mispositioned interneurons within the GCL (Fig. 5P–R). Surprisingly, immunohistochemical analysis for Calretinin in *Dab1^−/−^* animals (Fig. 5J–L) revealed only a weak phenotype at P7 and P14. No clusters were observed and the neurons showed oriented processes but with less strict orientation as seen in wild type animals. However, at adult stage the phenotype of *Dab1^−/−^* animals appeared similar than the one observed in wild type.

These observations suggest that one or maybe both of the Reelin receptors participate in Reelin-mediated layering. To clarify this question we analyzed Vldlr and ApoER*2* single KO animals for Calretinin expressing neurons. *Vldlr^−/−^* mutant mice appeared to be indistinguishable from wild type animals at all ages (Fig. 5D–F). In contrast, *ApoER2* ^−/−^ mutants showed one week after birth a similar aberrant morphology of Calretinin-positive neurons as in *reeler* and *ApoER2* ^−/−^:*Vldlr^−/−^* animals (Fig. 5G, M and P). However, at P14 the formation of a more compact granule cell layer starts with some cells extending their neurites towards superficial layers (Fig. 5H). At adult stage also *ApoER2* ^−/−^ mutants exhibit a minor phenotype reminiscent of *reeler* mice with some Calretinin positive interneurons that have lost orientation whereas the majority upholds a strict radial organization (Fig. 5I).

Investigating the Calretinin-positive cell population hosting in the GL no abnormalities were observed in the different mutants at adult stage (data not shown). These findings point towards ApoER2 as being the main receptor for mediating Reelin functions in the OB and particularly layer formation.

### The Radial Glial Scaffold is not Altered

Our findings suggest an influence of the Reelin signaling cascade on the migration of interneurons within the OB. We addressed next, whether the glial scaffold was altered in the different mutant animals, which could explain the interneuron migration and neurite orientation phenotypes. However, performing BLBP (brain lipid binding protein) immunohistchemistry at P0 did not reveal any alterations in the organization of the OB glia scaffold in *ApoER2^−/−^*, *Dab1^−/−^*, *reeler* and *ApoER2^−/−^:Vldlr^−/−^* animals (Fig. S1A–E).

### Vldlr mRNA Expression Increases with Age but is not Altered in ApoER2 Mutants

The morphological changes in the GCL (Fig. 5) suggest for a compensatory mechanism in *ApoER2^−/−^* mutants since the observed double receptor knockout phenotype is markedly attenuated after P7. In view of the canonical Reelin signaling pathway Vldlr is a possible candidate molecule to rescue the *ApoER2* ^−/−^ phenotype. Therefore, we examined *Vldlr* mRNA changes in *ApoER2^−/−^* mutant animals by quantitative RT-PCR. We did not observe any significant changes in the expression of *Vldlr* when comparing wild-type animals and *ApoER2^−/−^* mutant mice at P7 and P14, respectively (data not shown). However, comparison of *Vldlr* expression levels between P7 and P14 showed a small but significant 1.29-fold increase of *Vldlr* mRNA expression in *ApoER2^−/−^* animals from P7 to P14 and a 1.4-fold increase in wild type demonstrating an age dependent increase of *Vldlr* mRNA (data not shown).

### Collapsin Response Mediator Protein 1 Could Act Downstream of ApoER2 in the OB

We observed that *Dab1^−/−^* neuroblasts were clearly distinguishable from *reeler* and *ApoER2* ^−/−^:*Vldlr^−/−^* neuroblasts regarding detachment and migration in vitro (Fig. 3). Furthermore, adult *Dab1^−/−^* animals exhibited a normal granule cell layer. in contrast to *ApoER2* ^−/−^:*Vldlr^−/−^* and *reeler* mice (Fig. 5) Therefore, we asked whether other adapter molecules could mediate Reelin signaling via ApoER2 and Vldlr. Remarkably, it has been shown that Collapsin Response Mediator Protein 1 (Crmp1) mediates Reelin signaling in cortical neuronal migration [48]. In situ hybridization for Crmp1 on sagittal sections of adult wild-type animals (Fig. S2A, B, see also Allen Brain Atlas, http://mouse.brain-map.org, Ref. 73512561) revealed expression of Crmp1 in the rostral migratory stream in the MCL and in a periglomerular position. In mice deficient for Dab1, a similar expression of Crmp1 was found (Fig. S2C). Western immunoblotting showed increased expression levels for Crmp1 in membrane fractions of OB tissue of Dab1 mutants compared to wild-type animals (Fig. S2D) suggesting Crmp1 as being a candidate molecule for mediating ApoER2 and/or Vldlr signaling in the OB.

## Discussion

Studies on corticogenesis have provided evidence for different modes of neuronal migration [49], [50]. Besides somal translocation cortical neurons can use a glia-guided mode of migration to govern the increasing distance from the germinal ventricular zone to their definitive positions in the cortical plate [51]. This way, the layers of the cortex are formed in an inside-out manner [1], [52]. Since aberrant migration of glia-guided neurons has been reported in ApoER2 mutant mice a key role for ApoER2 in this process has been proposed [44].

Similar to cortical lamination layer formation in the OB takes place chronologically. First mitral cells as principal projection neurons are generated and provide a local Reelin source [6]. Subsequently, interneurons follow in a sequential order [2]. The calbindin positive subpopulations represent early generated interneurons. The majority of these interneurons originate from late embryogenesis and there is a marked decline in their generation immediately post-partum. In contrast, only few of the Calretinin-positive interneurons are produced embryonically, while the majority is produced after birth. Turnover of late-generated interneurons persists throughout adulthood [2], [6].

In analogy to cortical neuronal migration, glia-guided migration has been postulated for OB layer formation [53]. The data of our study are consistent with this idea. The aforementioned rapid decrease in glia is coincident with the timeframe of generation of Calbindin positive interneurons [2]. In the ApoER2 mutants but not the Vldlr mutant mice migration of the early generated Calbindin-positive subpopulations is disturbed similar to *reeler* and *dab^−/−^* mutants. Furthermore, we found that also early born TH-positive interneurons show migration defects in ApoER2 and ApoER2:Vldlr double receptor knock-out animals but not in Vldlr mutants. In accordance, two other observations are substantiating the idea that Reelin-ApoER2 signaling regulates early postnatal interneuron migration. Firstly, double-receptor-knockout mice exhibit the same misplacement of early born interneurons. Secondly, interneurons expressing Calretinin exhibit a strong phenotype in *ApoER2^−/−^* animals similar to *reeler* mice at early postnatal stages. However, this phenotype declines with development and adult animals show only minor alterations in the Calretinin population compared to wild type animals. These defects are not seen in *Vldlr^−/−^* mutants. Thus, our data support the model that ApoER2 signaling is involved in early Vldlr-independent migration, most likely glia guided [53]. Since we were unable to find obvious changes in the radial glial scaffold we assume that the interaction of the migrating neuron with the radial fiber is altered similar to the situation seen in the developing cortex in a yet unknown way [53].

A previous report claimed the absence of VLDLR protein in the mouse OB by immunohistochemical analysis [54]. In contrast, we identified expression of Vldlr protein by Western immunoblotting in the OB of adult mice and furthermore by RT-PCR and *in situ* hybridization studies in postnatal and adult animals. Based on these findings we concluded the presence of Vldlr in the postnatal and adult mouse OB. Therefore, compensatory mechanisms mediated by Vldlr in adult *ApoER2^−/−^* animals could be responsible for the decline and adjustment of the Calretinin phenotype. This idea is underlined by the fact that in adult ApoER2:Vldlr double knock out animals no compensation but a *reeler*-like phenotype can be observed within the Calretinin population of the GCL. Interestingly, we did not find defects in the migration of Parvalbumin positive interneurons in any of our mutant animals. Since the majority of this population shows migration after Calbindin- but before Calretinin-positive interneurons we assume that this population migrates independent of the Reelin signaling cascade. Similarly, Tbr1- and Reelin-positive mitral cells. These principle neurons are already present at prenatal stages (E13) and likely migrate in a Reelin-independent fashion.

Late generated Calretinin-positive interneurons are continuously replaced throughout adulthood. Detachment of chain migrating neuroblasts from the rostral migratory stream is accompanied by the induction of a leading process, which directs radial individual migration of Calretinin positive interneurons towards their final destination [19]. Here, Reelin has been shown to function as detachment factor [36] either by acting on the interaction between radial processes and migrating neurons or on the interaction between apposing cells in chain migration [19]. However, we show that in vitro the Reelin detachment signal for neuroblasts is mediated by ApoER2. Absence of Vldlr and surprisingly also the intracellular adapter protein Dab1 did not influence the detachment process. To analyze whether these findings are also seen in vivo we analyzed first the presence and the functional integrity of the RMS in all our mutants. We found that all adult mutant strains have an RMS and showed BrdU-positive cells within the OB 16 day after peritoneal injection of BrdU. Hence, neuroblasts derived from the SVZ arrive and integrate in the OB of mutant mice arguing for the presence of a functional RMS. Although ApoER2, Vldlr and Dab1 are expressed within the RMS, canonical Reelin signaling seems not to be pivotal for RMS function [40]. However, our immunohistochemical results revealed a disruption of the GCL morphology in *ApoER2* ^−/−^, *reeler* and double receptor knockout mice one week after birth. Since Vldlr^−/−^ animals do not show this phenotype at early postnatal stages these data support our *in vitro* results that ApoER2 regulates migration of Calretinin-positive neurons. Furthermore, in adult *reeler* and double receptor knockout mice, PSA-NCAM positive neuroblasts do not possess a leading process, a prerequisite for correct migration. Moreover, most cells lose their radial orientation and accumulate in clusters. However, with advancing age the migration defect of Calretinin-positive interneurons in ApoER2 mutants attenuates and we observed correct detachment of precursor cells in adult *ApoER2* ^−/−^ and Vldlr^−/−^ mice including leading process induction. Thus, we propose redundant function for Vldlr and ApoER2 in the late postnatal and adult OB. The following aspects point towards this. Firstly, the expression pattern of Vldlr changes. While the RMS of early postnatal animals is *Vldlr* negative, migrating neuroblasts in the adult OB express *Vldlr* mRNA. Secondly, the *Vldlr* expression level increases within the critical time between P7 and P14. Thirdly, the disruption of GCL morphology persists in double receptor knockout mice, which resembles the *reeler* phenotype.

Dab1^−/−^ mutant mice do not exhibit a detachment phenotype in our *in vitro* model. Furthermore, *in vivo*, the detachment process appears to be only slightly impaired similar to the formation of the GCL. Although BrdU incorporation in outer layers of the OB was affected in Dab1^−/−^ animals, no effect was observed in the core region in the vicinity of the RMS. However, although the loss of Dab1 could affect neuronal migration in a non-cell autonomous way since Dab1 is also expressed by glia [55], our results suggest also for a compensatory mechanism for Dab1 downstream of the lipoprotein receptors. Therefore, we analyzed the expression of candidate molecules and found Crmp1 as being very similar expressed in the adult forebrain as Dab1. Similar to Dab1, Crmp1 has been described as an intracellular signaling mediator of Reelin during corticogenesis [48]. Based on its expression, Crmp1 could also play a role in mediating Reelin signaling in the OB and therefore being a candidate molecule responsible for the mild phenotypes seen in Dab1 mutant animals in the OB. However, future studies will be required to clarify this hypothesis.

Canonical Reelin and lipoprotein receptor signaling appear to be highly redundant and compensatory mechanisms seem to account for partially contradictionary results obtained in studies using knock-out animal models (see e.g. study by Andrade et al. [40] and the presented data). Therefore, it might be important for future studies to emphasize genetic background variations in individual mouse strains when comparing different mutant animals since background variations are known to influence redundant signaling pathways.

## Supporting Information

Figure S1 **Radial glial scaffold in the OB.** Frontal sections through the OB of wild-type (A), *ApoER2^−/−^* (B), *reeler* (C), *ApoER2^−/−^:Vldlr^−/−^* (D) and *Dab1^−/−^* (E) mice at P0 stained for BLBP. No alterations were observed. GL, glomerular cell layer; EPL, external plexiform layer; MCL, mitral cell layer; GCL, granule cell layer. Scale bar: 50 µm.
(TIF)

Figure S2 **Crmp1 expression in the forebrain.** In *situ hybridization* on sagittal brain sections revealed expression of *crmp1* mRNA in the rostral migratory stream, the mitral cell layer and in a periglomerular position in wild-type mice (**A, B**) and Dab1^−/−^ mutants (**C**). (**D**) Western immunoblotting analysis of the membrane fraction of OB tissue at P21 showed an up-regulation of Crmp1 expression level in of Dab^−/−^ mice compared to wild-type animals. Blots were obtained with similar amounts of proteins as indicated by the immunoblot for ß-actin. Molecular weights are shown on the right of each panel. Scale bar: 100 50 µm.
(TIF)

Figure S3 **Labeling of early generated TH-positive interneurons.** Immunohistochemistry for TH on frontal sections through the OB of adult wild-type mice showed strong expression in the glomerular cell layer (A, GL, wt). A similar staining was observed in *Vldlr^−/−^* mice (A, *Vldlr^−/−^*) although some cells were hosted in the external plexiform layer (arrowhead*)*. *ApoER2^−/−^* and *ApoER2* ^−/−^:*Vldlr^−/−^* mutants show clearly two separate cell populations (A). A typical superficial periglomerular labeling and a deep layer staining in the EPL (A, arrowheads). (B) Quantification of the relative number of TH positive cells per area in the EPL after normalization to the wt situation (n = 3–5 animals per genotype) shows a significant quantitative mispositioning of TH-positive neurons in the EPL in *ApoER2^−/−^* and *ApoER2* ^−/−^:*Vldlr^−/−^* mutants but not in *Vldlr^−/−^* animals. Wilcoxon-Mann-Whitney test; Data expressed as mean ± SEM. Scale bar 100 µm.
(TIF)
