# Supplementary figures and images for: Reelin Together with ApoER2 Regulates Interneuron Migration in the Olfactory Bulb
Source: PLoS One. 2012 Nov 29;7(11):e50646. doi: 10.1371/journal.pone.0050646 (PMC3510185; doi:10.1371/journal.pone.0050646)

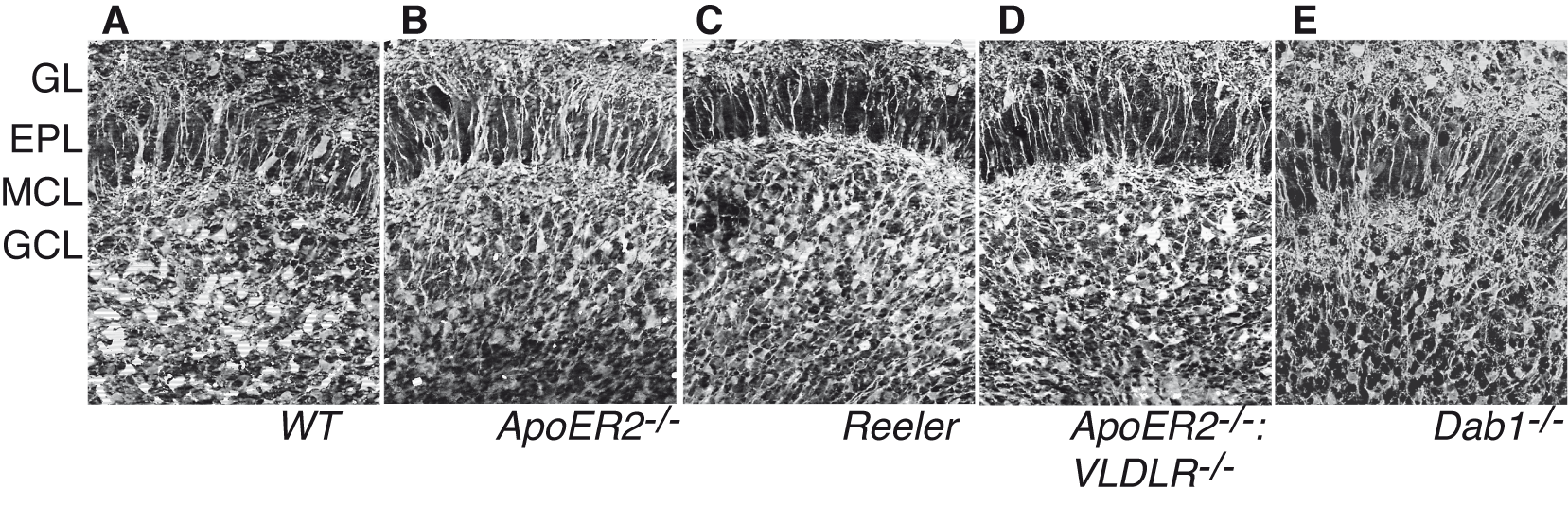

Supplement: Figure S1 — Radial glial scaffold in the OB. Frontal sections through the OB of wild-type (A), ApoER2−/− (B), reeler (C), ApoER2−/−:Vldlr−/− (D) and Dab1−/− (E) mice at P0 stained for BLBP. No alterations were observed. GL, glomerular cell layer; EPL, external plexiform layer; MCL, mitral cell layer; GCL, granule cell layer. Scale bar: 50 µm. (TIF) [file pone.0050646.s001.tif]

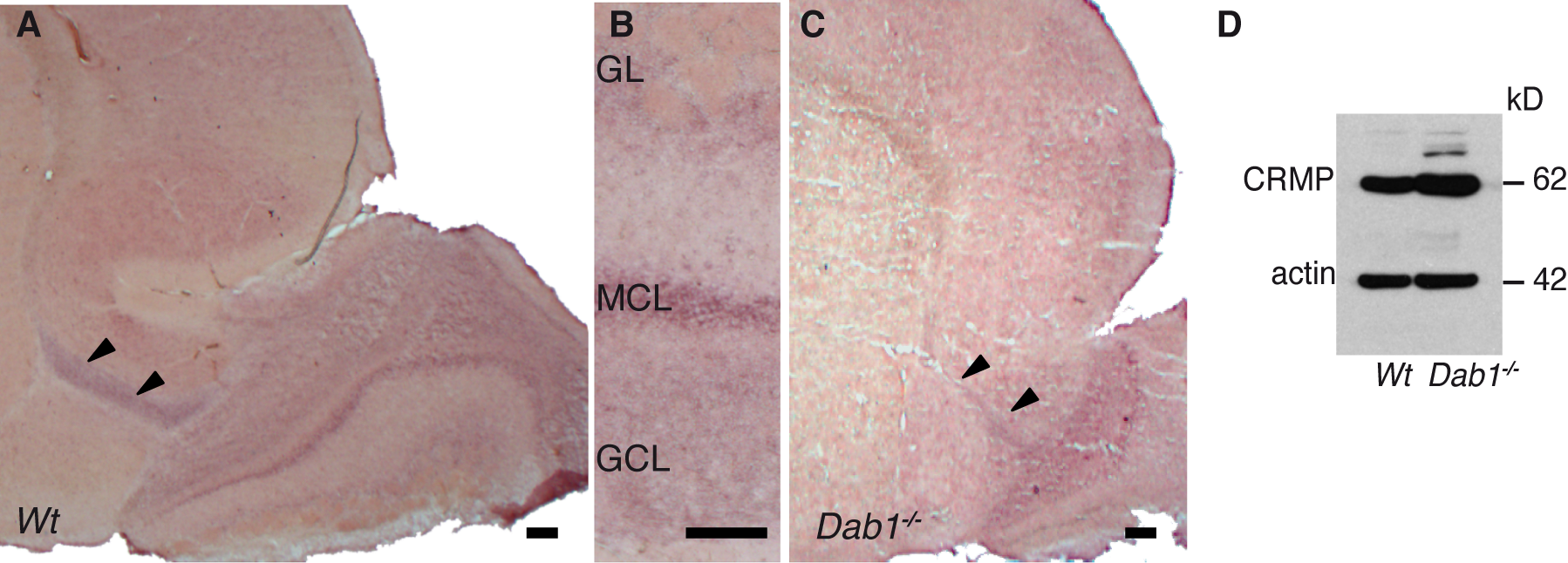

Supplement: Figure S2 — Crmp1 expression in the forebrain. In situ hybridization on sagittal brain sections revealed expression of crmp1 mRNA in the rostral migratory stream, the mitral cell layer and in a periglomerular position in wild-type mice (A, B) and Dab1−/− mutants (C). (D) Western immunoblotting analysis of the membrane fraction of OB tissue at P21 showed an up-regulation of Crmp1 expression level in of Dab−/− mice compared to wild-type animals. Blots were obtained with similar amounts of proteins as indicated by the immunoblot for ß-actin. Molecular weights are shown on the right of each panel. Scale bar: 100 50 µm. (TIF) [file pone.0050646.s002.tif]

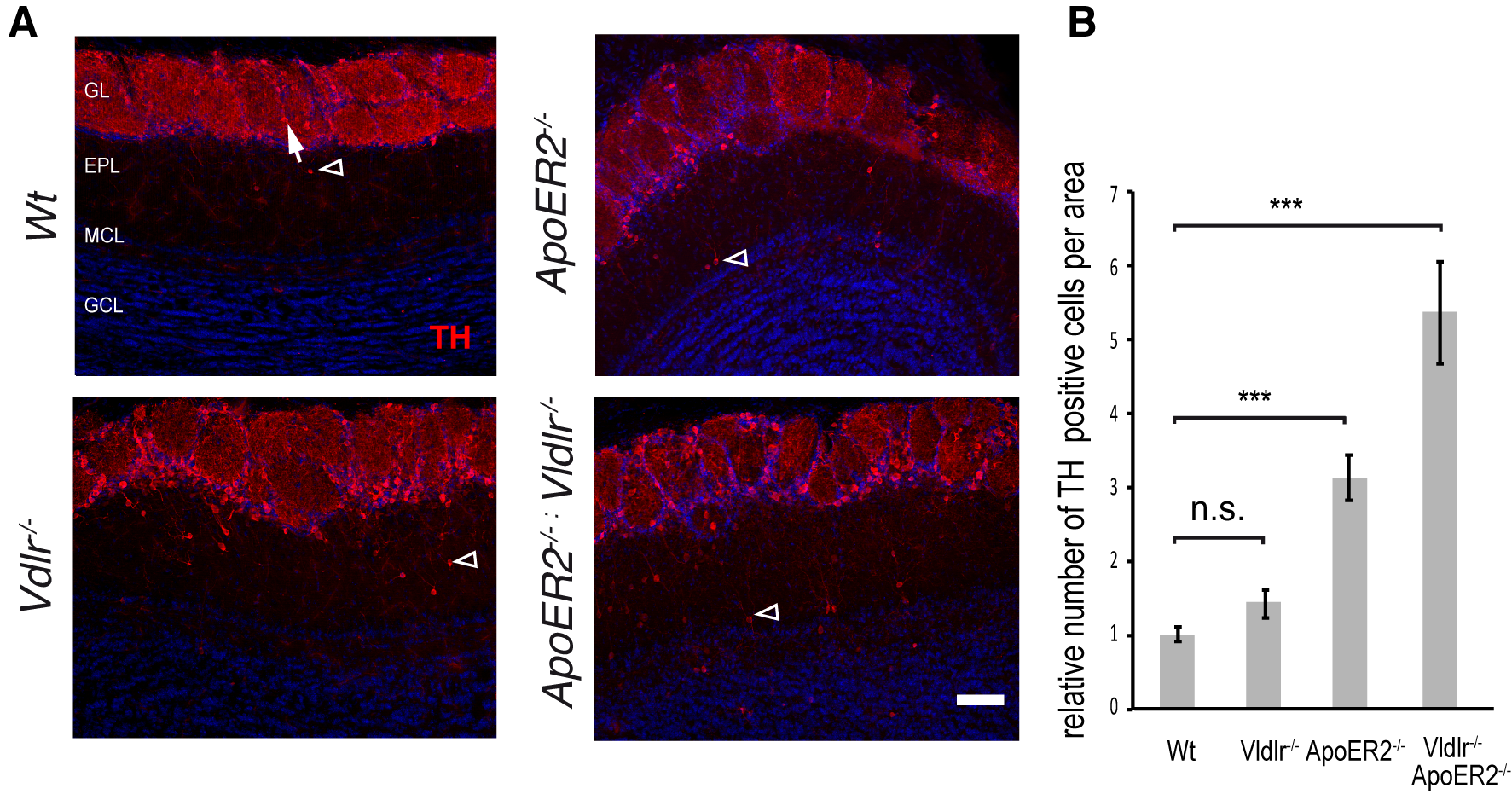

Supplement: Figure S3 — Labeling of early generated TH-positive interneurons. Immunohistochemistry for TH on frontal sections through the OB of adult wild-type mice showed strong expression in the glomerular cell layer (A, GL, wt). A similar staining was observed in Vldlr−/− mice (A, Vldlr−/−) although some cells were hosted in the external plexiform layer (arrowhead). ApoER2−/− and ApoER2 −/−:Vldlr−/− mutants show clearly two separate cell populations (A). A typical superficial periglomerular labeling and a deep layer staining in the EPL (A, arrowheads). (B) Quantification of the relative number of TH positive cells per area in the EPL after normalization to the wt situation (n = 3–5 animals per genotype) shows a significant quantitative mispositioning of TH-positive neurons in the EPL in ApoER2−/− and ApoER2 −/−:Vldlr−/− mutants but not in Vldlr−/− animals. Wilcoxon-Mann-Whitney test; Data expressed as mean ± SEM. Scale bar 100 µm. (TIF) [file pone.0050646.s003.tif]
